# Contribution of BCR-ABL molecular variants and leukemic stem cells in response and resistance to tyrosine kinase inhibitors: a review

**DOI:** 10.12688/f1000research.74570.1

**Published:** 2021-12-15

**Authors:** Mohammad Al Hamad

**Affiliations:** 1 Department of Pathology, College of Medicine, Imam Abdulrahman Bin Faisal University, Dammam, Dammam, 31441, Saudi Arabia

**Keywords:** Chronic myeloid leukemia, BCR-ABL, Kinase domain mutations, Leukemic stem cell, Tyrosine kinase inhibitors, mi RNA.

## Abstract

Chronic myeloid leukemia (CML) is a myeloproliferative neoplasm generated by reciprocal chromosomal translocation, t (9; 22) (q34; q11) in the transformed hematopoietic stem cell. Tyrosine kinase inhibitors (TKIs) target the mature proliferating BCR-ABL cells, the major CML driver, and increase overall and disease-free survival. However, mutant clones, pre-existing or due to therapy, develop resistance against TKIs. BCR-ABL1 oncoprotein activates various molecular pathways including the RAS/RAF/MEK/ERK pathway, JAK2/STAT pathway, and PI3K/AKT/mTOR pathway. Stimulation of these pathways in TKI resistant CML patients, make them a new target. Moreover, a small proportion of CML cells, leukemic stem cells (LSCs), persist during the TKI therapy and sustain the disease in the patient. Engraftment of LSCs in the bone marrow niche and dysregulation of miRNA participate greatly in the TKI resistance. Current efforts are needed for determining the reason behind TKI resistance, identification, and elimination of CML LSC might be of great need for cancer cure.

## Introduction

The BCR-ABL gene results from a reciprocal translocation t (9; 22) (q34; q11) in the transformed hematopoietic stem cell (HSC). ^1^^–^^3^ BCR-ABL is considered the most common genetic abnormality in chronic myeloid leukemia (CML) (95%), followed by acute lymphocytic leukemia (ALL) (35%), and rarely presented in acute myelogenous leukemia (AML) (1%). ^4^^–^^6^ The most detected variant of BCR-ABL in CML is P210 BCR-ABL1 that occasionally presents in ALL and AML. The P210 BCR-ABL1 variant expresses in about 48% of Ph+ CML cases, while 52% co-express P210 BCR-ABL1 and P190 BCR-ABL1 variants. ^7^ Approximately 50% of CML patients present without symptoms and are diagnosed incidentally after routine laboratory tests. ^8^ Cytogenetics is the standard diagnostic tool of CML; the Ph chromosome is detected in 90% of the cases. However, 5% of CML cases have a cryptic Ph chromosome that could not be detected by karyotyping. The cryptic Ph chromosome can be detected by fluorescence *in situ* hybridization (FISH) and/or real-time polymerase chain reaction (PCR). ^9^

Clinically CML is divided into three different stages: chronic phase (CP), accelerated phase (AP), and blast phase (BC). Without intervention treatment, the disease progresses from the chronic phase to blast phase which is most likely accompanied by multiple genomic abnormalities. Here we present a review of the BCR-ABL response and resistance to TKI therapy.

## BCR-ABL gene & protein

BCR-ABL results in the juxtaposition of 3′ sequences of ABL (Abelson proto-oncogene) on chromosome 9 and the 5′ sequences of the truncated BCR (breakpoint cluster region) on chromosome 22. Consequently, the BCR/ABL fusion gene forms on chromosome 22 that encodes BCR-ABL1 oncoprotein, a constitutively active tyrosine kinase. ^10^^–^^12^

The constitutive tyrosine kinase activity of the chimeric BCR-ABL1 oncoprotein, deregulates the downstream signaling of many pathways which results in uncontrolled proliferation, arresting the differentiation of the HSC, and limits apoptosis. Consequently, the normal HSC transforms into leukemic stem cells (LSC), which accumulates in the bone marrow (BM) and thus replaces the normal stem cells. ^13^^,^^14^

Three different BCR-ABL chimeric proteins emerge from the fusion of mRNA molecules of different lengths of the ABL gene with the BCR gene. The most common breakpoint in BCR occurs in intron 13 or intron 14, then exon 13, or 14 exons (M-BCR) fused to the ABL1 gene at exon a2, which is referred to as e13a2, e14a2. These fusions result in a BCR-ABL1 protein with 210 kilodaltons molecular mass (p210 BCR-ABL1), present in 95% of CML. The second alternative (<1%) is that the e19a2 fusion transcript produces a larger fusion protein with 230 kilodalton weight (p230 BCR-ABL1), a diagnostic marker for neutrophilic-chronic myeloid leukemia. The third alternative is p190 BCR-ABL1 protein resulting from e1a2 fusion transcript, ^6^ most common in B cell ALL, less so in AML, and rarely presented in CML.

BCR-ABL oncoprotein possesses different domains from BCR and ABL. The BCR portion includes the NH2-terminal coil-coil (CC) domain (amino acid 1-63), an oligomerization domain, followed by a serine/threonine kinase domain-containing Tyr 177, a binding site for growth factor receptor-binding protein 2 (GRB2), and Ras homolog gene/guanine nucleotide exchange factor (Rho/GEF) kinase domain, a GTPase activating protein that gets activated when binded to GTP and turned off when binded to GDP, and thus control the activation of Ras protein. Whereas ABL domains include Src- homology (SH) SH3, SH2, and SH1 (Kinase) domains, followed by proline-rich domain and binding domain (BD), and facilitate the nuclear protein, actin, and DNA binding. CC domain and Y177 are required for the efficient activation of ABL kinase. Tetramerization of BCR-ABL is essential for constitutive kinase activity. Targeting the CC domain deregulates the tetramerization of BCR-ABL, reduces the kinase activity, and increases sensitivity to imatinib (tyrosine kinase inhibitor). ^15^^,^^16^

In BCR-ABL, Y177 plays a crucial role in leukemic progenitor cell expansion, proliferation, and survival through activation of the Ras and PI3K/Akt pathway. Interestingly, mutated 177Y fail to develop CML and increase sensitivity to imatinib, suggesting a target of Y177 to eliminate the leukemic stem cells. ^17^ Rho/GEF retained in p230 and p210 BCR-ABL but not in p190, thought to facilitate calcium-dependent lipid binding, restrict proliferation and support survival. ^18^

ABL protein has a tyrosine kinase function that is regulated by SH2 and SH3 domains in the N-terminal end. SH3 is considered as a negative regulator of the tyrosine kinase activity, therefore inhibition of SH3 leads to an abnormal increase in the enzyme activity and enhances the malignant transformation. ^19^

The SH1 domain of ABL has an autophosphorylation site that activates kinase as conjugates to ATP. However, ATP-binding inhibitors, compete with ATP on its binding site and impeding the downstream intracellular signaling pathway. ^20^^,^^21^ SH2, the protein-protein interaction domain, activates the SH1 by establishing a tight junction with the N-terminal lobe of SH1, resulting in downstream activation of the intracellular signaling pathways. Interestingly, the open conformation of SH1 allows interacting with the SH2 domain, whereas the close conformation prevents this binding. The SH3 of ABL binds to SH1 through N-terminal myristoyl modification, which induces conformational changes on SH1 that allow SH2-SH3 docking onto it. ^22^

In a normal physiological state, tyrosine kinase (TK) regulates the activities of the cell cycle. Constitutive activation of TK interrupts the normal state of the cell cycle, resulting in inhibition of cell differentiation, uninterrupted activation of the cell proliferation, and escapes cell apoptosis. Thus, BCR-ABL deregulates many pathways. ^23^

ABL protein shuttles between the nucleus and cytoplasm; however, the chimeric BCR-ABL oncoprotein stops this shuttle and retains ABL protein in the cytoplasm, where it interacts with many different proteins that are involved in the malignant transformation. ^24^

In BCR-ABL fusion, the N-terminal oligomerization domain of BCR activates the ABL tyrosine kinase by blocking the SH3 on the N-terminal of ABL1. ^25^ Accordingly, BCR-ABL oncoprotein activates diverse signaling pathways including the RAS pathway, JAK2/STAT pathway, and PI3K-AKT-mTOR pathway. BCR-ABL oncoprotein directly activates JAK2/STAT pathway and continuously enhances cell survival in CML. ^26^

## Response and resistance to TKI

TKIs have dramatically changed the treatment and prognosis of CML. TKI targets the proliferating mature BCR-ABL cells which are considered the major driver of CML. However, a minority of subclones with genetic aberration and quiescent leukemic stem cells are not eliminated by TKI. ^27^^,^^28^ Molecular resistance is measured by quantitative real-time polymerase chain reaction (qRT-PCR) of BCR-ABL1 and cytogenetic resistance (Ph+ persistence). These procedures are considered the main standard for monitoring the patient response to therapy, predicting relapse, and guide treatment decisions. ^29^

Binding of ATP to tyrosine kinase active site of BCR-ABL oncoprotein phosphorylate tyrosine residue of substrate results in progression of CML. TKI impedes the ATP- tyrosine kinase binding and thereby halts the constitutive tyrosine kinase activity of the BCR-ABL1 oncoprotein and thus inhibits the CML progression. There are three types of TKI inhibitors. Type I is ATP competitive inhibitors like imatinib and nilotinib which compete with ATP in the ATP binding site of the kinase domain. This, consequently, prevents autophosphorylation, substrate phosphorylation and thus inhibits proliferation and induces apoptosis for BCR-ABL cells. Type II is ATP competitive inhibitors like dasatinib, which bind to the ATP-binding site as well as the hydrophobic adjacent binding site which is accessible only when the kinase is in an inactive configuration. ^30^ The third type is allosteric inhibition which involves the binding of TKI to the allosteric-myristate binding pocket, induces a conformational change in the kinase domain and renders the kinase-inactive. ^31^ It has been estimated that about 25% of CML patients develop resistance to TKI that necessitates to switch TKIs at least once during their lifetime. ^32^

Resistance to imatinib occurs in approximately 17% of patients with 5 years of follow-up. The mechanism of TKI resistance is subdivided into ABL1 mutation-dependent (acquired resistance) which means loss of response, and ABL1 mutation-independent (primary resistance), which results in conformational changes of the TKI binding site due to mutation in SH1 of ABL1. ^33^ Therefore, a new target therapy or combining TKI is required to cure CML. As a result, the new version of TKIs, dasatinib, and bosutinib, have been approved as first-line therapy for imatinib resistance patients. ^34^

**Figure 1.**
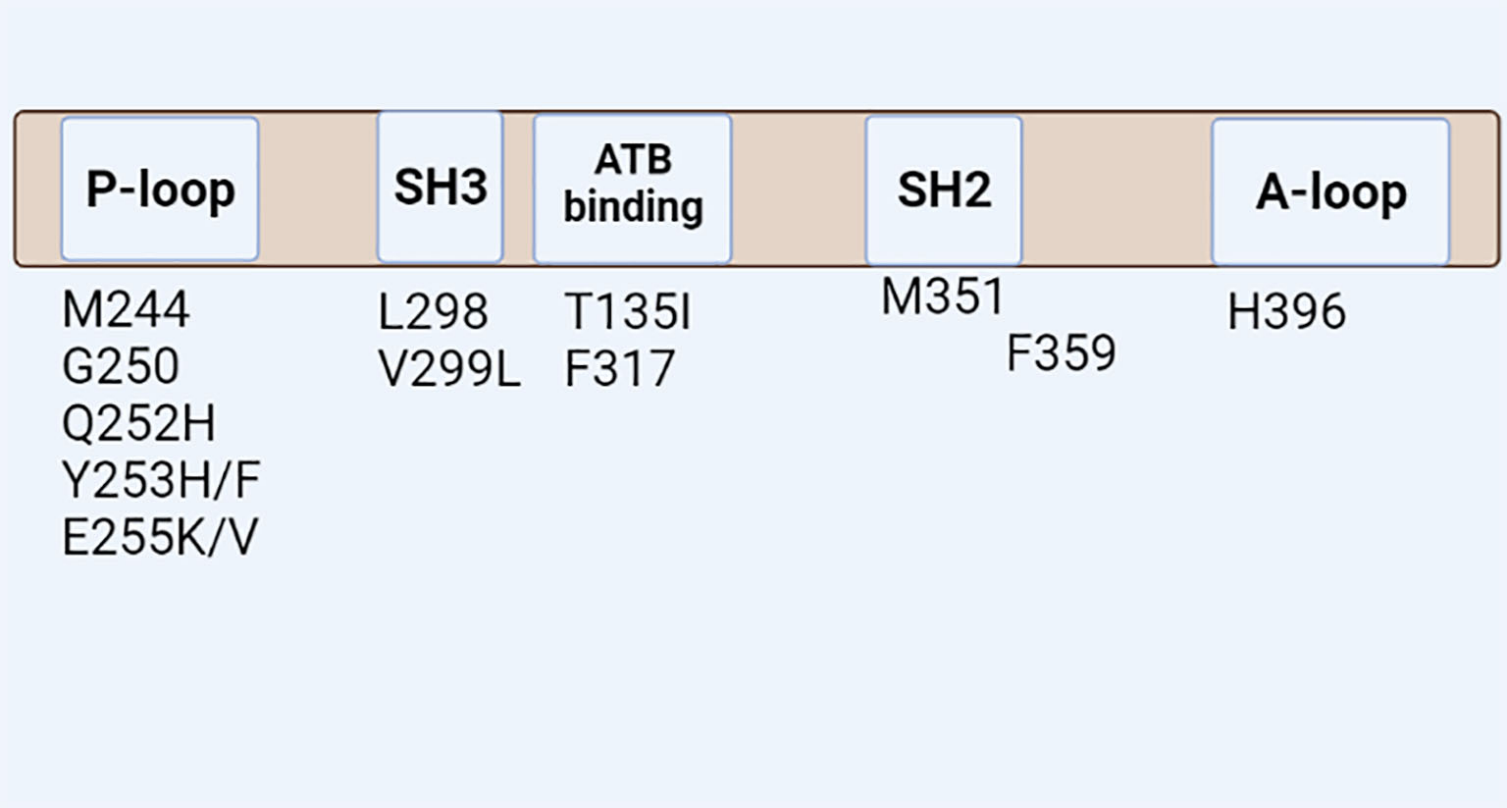
Schematic demonstrates the types of ABL (Abelson proto-oncogene) kinase mutations.

**Figure 2.**
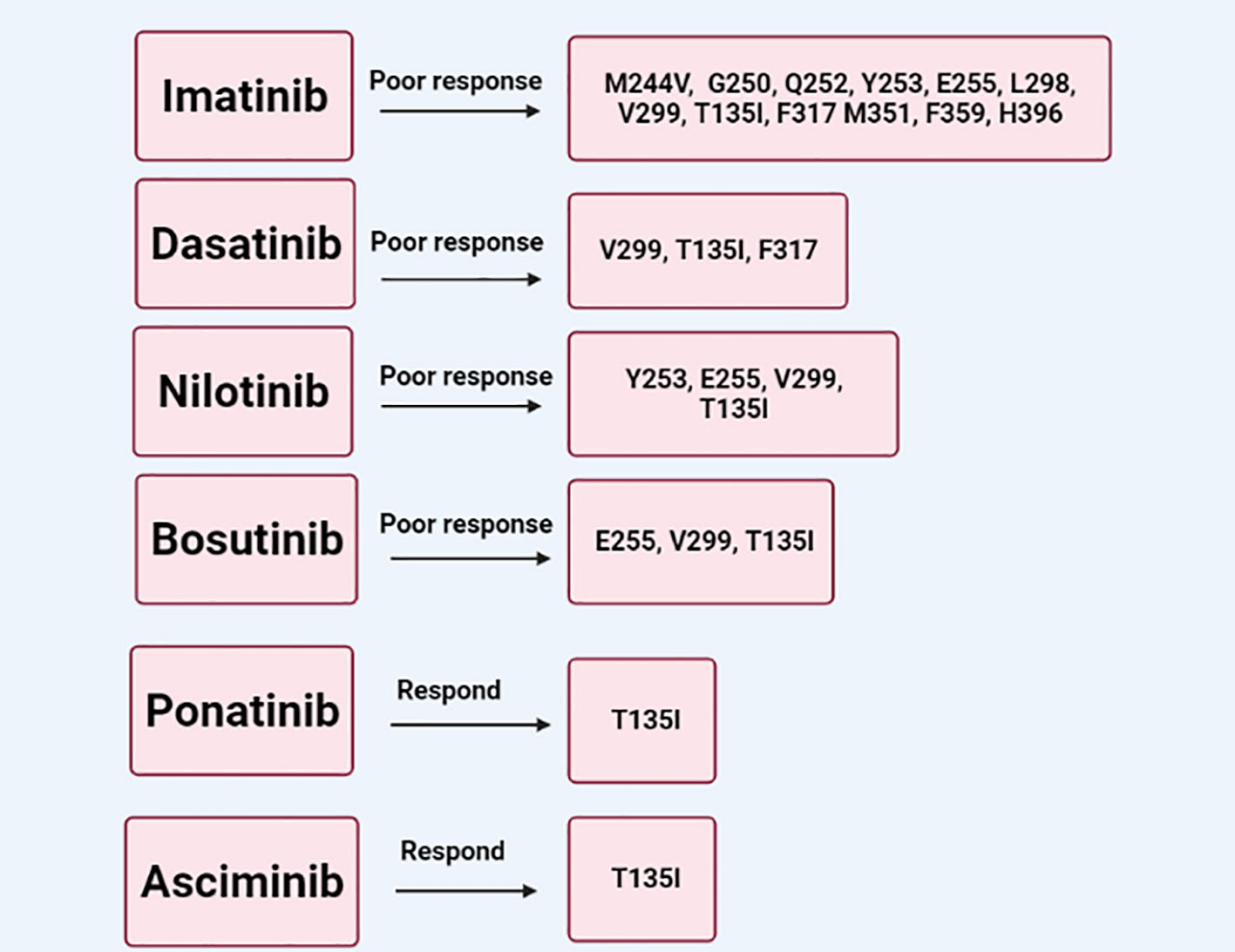
Schematic demonstrates the types of kinase domain mutations with their response and resistance to imatinib, dasatinib, nilotinib, bosutinib, ponatinib, and asciminib.

The dominant mechanism of imatinib resistance is acquired mutation in the kinase domain (KD) of BCR-ABL with greater frequency in AP, BP CML patients than CP. ^35^^,^^36^ These mutations reside in phosphate binding loop at positions M244, G250, Q252H, Y253H/F, and E255K/V, gatekeeper (T315, F317), the activation lobe (H396), SH2, and C-lobe (M351, F359) that ultimately impaired TKI binding by affecting the essential residue for direct contact or by preventing the inactive conformation of ABL kinase domain appropriate for imatinib binding ^37^ ( Figure 1). Nilotinib shares the same binding site of imatinib with significantly higher affinity that overcomes many imatinib-resistant mutations except for Y253, E255, T315I, and F359 mutations. ^38^^,^^39^ About 25% of CML patients develop T315I mutation that inhibits TKI binding and develop resistance in all TKIs except ponatinib. ^40^ T315I mutation, which occurs in gatekeeper residue of the ABL domain, affects the binding affinity of imatinib, dasatinib, bosutinib, and nilotinib to the ATP-binding site. ^34^^,^^38^ Mutations within the ABL kinase domain and overexpression SRC are among the imatinib resistance mechanisms in CML. Dasatinib and bosutinib have dual inhibitory action against SRC and ABL kinase that address most of the BCR-ABL mutations associated with TKI resistance except T315I. ^41^^,^^42^ Ponatinib, a pan-BCR-ABL inhibitor, has a great efficacy against native and mutated BCR-ABL1 including the T315I variant. ^43^ However, a dose-dependent increased risk of developing arterial occlusion, stroke, limb ischemia, and other vascular events has been found. ^44^^,^^45^ Asciminib is an allosteric inhibitor, unlike other TKI inhibitors, that binds the myristoyl pocket of BCR-ABL. Asciminib is a potent and highly selective inhibitor of both native and mutated BCR-ABL including the gatekeeper T315I mutant. Asciminib is considered a relatively safe option for CP or AP CML with resistance to the currently available TKIs ^46^^,^^47^ ( Figure 2).

BCR-ABL-independent resistance might develop through upregulation of the PI3K/AKT/mTOR pathway. ^48^ At this point, PI3K/AKT/mTOR pathway inhibitors might be a new target for TKI resistance in CML patients. ^49^ NVP-BEZ235 is a dual inhibitor which shows efficacy against imatinib-resistance CML by arresting the cell cycle at G0/G1 and inactivating the PI3K/AKT/mTOR pathway. ^50^

## CML leukemic stem cells

CML leukemia stem cells (LSCs) are subpopulations of CML cells that persist through TKI therapy and sustain the disease in patients. LSCs are quiescent cells that reside in the microenvironment of BM and remain a potential reservoir for disease relapse. LSCs are characterized by high heterogeneity of genetic, epigenetic, and transcriptional mechanisms. ^51^ The persistent LSCs result from resistance to targeted therapy that might happen primarily as a lack of initial response to treatment, or acquired as disease relapses after initial response to the therapy. ^52^ Of crucial importance, determination, and elimination of LSC might be of great need for cancer cure.

**Figure 3.**
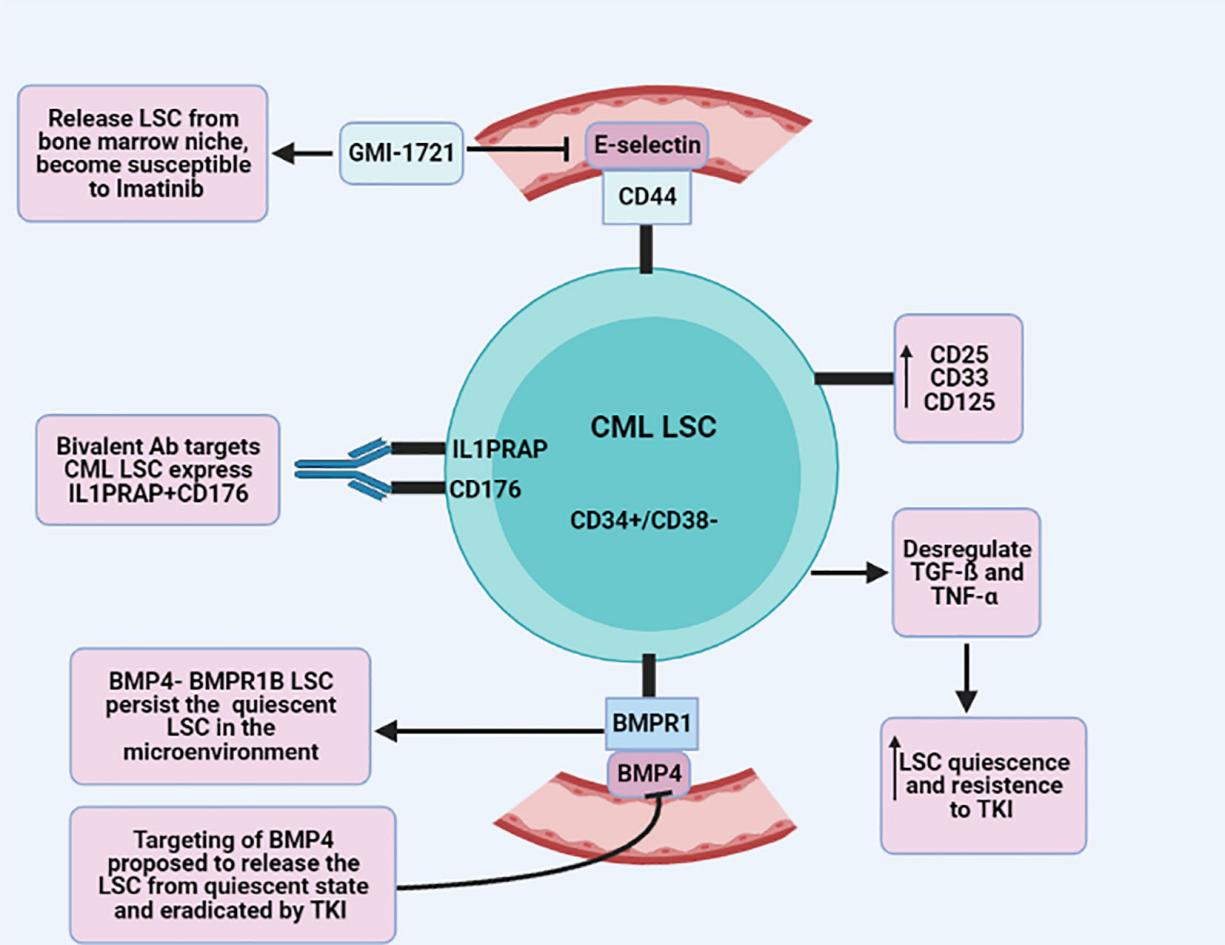
Schematic illustrates various mechanisms of CML-LSC (chronic myeloid leukemia-leukemic stem cell) resistance and response to TKI (tyrosine kinase inhibitor).

CD34+/CD38- is a stem cell marker of both normal stem cells and LSC. ^53^ CD34+/CD38- LSC in CML patients was found to express higher levels of CD33 compared to normal CD34+/CD38- stem cells. ^54^ Recently, Herrmann *et al*. demonstrated that CD34+/CD38- CML-LSC express higher levels of CD 25, CD33, and CD 125 compared to normal BM stem cells. Moreover, immunological targeted therapy against these markers resulted in depletion of CML-LSC and reduce LSC engraftment. ^55^ In a study of single-cell gene expression analysis combined with an immunophenotypic screening of CML-LSC, an aberrant expression of CML-LSC surface markers CD25, CD26, and interleukin1 receptor accessory protein (IL1RAP) was detected. These markers were found to be downregulated after TKI administration. ^52^ In contrast to normal stem cells, IL1RAP is a tight marker of BCR-ABL positive cells, and CD176 is a hematopoietic stem cell marker, co-expressed on CD34+/BCR-ABL+ cells of peripheral blood from CML patients. Interestingly, a bivalent antibody specific to both IL1PRAP and CD176 was found to target the cell population of CML but not their corresponding normal cells. ^56^ Another study demonstrated that BCR-ABL CML CD34+/CD38-/CD26+LSC cells are resistant to TKI. ^57^ Interestingly, it turned out that the pathways of TGF-ß and TNF-α were dysregulated in both BCR-ABL- and BCR-ABL+ CML-LSC with increased LSC quiescence and resistance to TKI. Moreover, increased serum levels of TGF-ß and TNF-α at diagnosis were associated with low treatment response in CML that might have an important role as a clinical predictive biomarker. ^52^ Codavarthy *et al*. demonstrated that expression of CD44 on BCR-ABL + LSC and E-selectin on bone marrow endothelium participates greatly in engraftment of LSC in bone marrow niche which protects the LSC from imatinib treatment. Interestingly, GMI-1271, an E-selectin inhibitor, showed that releasing of BCR-ABL+ from the bone marrow niche increase responsiveness to Imatinib by decreasing the leukocyte counts and BCR-ABL cells. ^58^ Bone morphogenetic proteins (BMP) are soluble growth factors that regulate stem cell fate and proliferation. The concentration of BMP2 and BMP4 was found to be higher in the bone marrow of CML patients in CP corresponding to normal bone marrow donors. ^59^ BMP bind to its receptor, BMP receptor type B1 (BMPR1B), on LSC in the tumor niche and thus sustain the survival of BMPR1B+ LSC and progenitor cells. Upon treatment, LSC and progenitor cells increase expression of BMPR1B, which progressively activate the BMP4 autocrine loop and result in TKI resistance. ^60^ The bi-directional signal of BMP4- BMPR1B LSC alongside the Jak2-stat pathway has a crucial role in the persistence of quiescent LSC in the microenvironment. Targeting BMP and the Jak2-stat pathway has been proposed to release the LSC from quiescent state and eradicate it by TKI ^58^ ( Figure 3).

## miRNA role in CML TKI-resistance

miRNA is a small non-coding single-stranded RNA that controls gene expression. ^61^ Normally, miRNA-126 is expressed at a high level in hematopoietic stem cells and progenitor cells which conserve quiescence and restrain cell cycle progression. An increased level of miRNA-126 support the quiescent and engraftment of CML LSC in the bone marrow niche. However, its level found to be downregulated in CML LSC compared to normal hematopoietic stem cells. Although BCR-ABL downregulates miR-126 in CML LSC, endothelial cells of bone marrow express a high level of miR-126 that supports CML LSC and thus sustain quiescence and leukemic growth. ^62^ Moreover, quiescent CML LSC expresses a higher level of miR-126 and more engraftment capacity than proliferating CML LSC. Interestingly, a combination of TKI and the CpG miR-126 inhibitor resulted in the elimination of LSC. ^61^

miRNA-409-5p expression is lower in peripheral blood of CML compared to healthy children. NUP43, the targeted gene of miRNA-409-5p, has shown to be overexpressed in CML children’s patients. ^63^ Overexpression of miRNA-409-5p downregulates the expression of NUP43 thereby inhibiting the proliferation and arresting the cell cycle. Furthermore, overexpression of miRNA-409-5p improves imatinib-induced proliferation inhibition and cell cycle arrest. ^63^ Recently, in a study of miRNome profiling of LSC from CML-CP patients revealed a nine-fold increase of miR-196a-5p in CD34+CD38−CD26+ (BCR-ABL+) compared to CD34+CD38−CD26- (BCR-ABL-). ^64^ Amplification of BCR-ABL and increased Jak2 signaling activate ADAR1, a double-stranded RNA protein that mediates adenosine to inosine (A-to-I) editing, thereby enhance the capacity of CML-LSC renewal by impairing the biogenesis of the Let-7miRNA family. ^65^

miR-451, a tumor suppressor, has demonstrated downregulation in some cancer types. In CML, miR-451 expression is associated with favorable prognosis, as this miR targets ABL and BCR-ABL directly. ^66^ In contrast, miR-21 is abundantly expressed in various solid and hematologic tumors. ^67^ Investigating miR-21 and miR-451 levels at diagnosis of CML is of great importance as a predictor of optimal TKI response. A high level of miR-451 at diagnosis was significantly correlated to optimal response to TKI after 6 and 12 months whereas a high level of miR-21at diagnosis predicts a lower probability of reaching the optimal response to therapy. ^68^ Inhibitors of miR-21 and PI3K together with imatinib significantly decrease AKT phosphorylation and C-MYC expression, thereby enhancing imatinib-induced apoptosis in CML-LSC. ^69^ Pellicano *et al*., demonstrated that has-miR183 is highly expressed in the BCR-ABL dependent pathway that mediates inhibition early growth response 1 (EGR1) leading to upregulation of E2F1, transcription factor, and consequently, enhance the proliferation of CML-LSC. ^70^ CML patients express a low level of miR30a compared to normal control individuals. Also, decreased level of miR30a has revealed an increase in the level of BCR-ABL that consequently enhances cell proliferation. ^71^ Moreover, targeting miR30a enhances imatinib activity against CML mediated by Becline1and autophagy protein 5. ^72^ Upregulation of miR29a-3p and miR660-5p target and downregulate *TET2* and EPAS1, respectively, that in turn confer TKI-resistance to CML-LSC. Furthermore, downregulation of miR-494-39 in LSC induces c-MYC overexpression, which is strongly connected to TKI-resistance BCR-ABL. ^73^

## Conclusion

TKIs have completely changed the treatment and improved the overall survival of CML patients. Dasatinib and bosutinib, new versions of TKIs, are considered as first-line therapy for CML patients who develop resistance to TKI (imatinib) as a result of KD mutation. CML LSC persists through TKI therapy and sustains the disease in patients. So, the determination and elimination of LSC might be of great need for cancer cure. Moreover, engraftment of CML LSCs in the bone marrow niche protect them from imatinib treatment. Therefore, inhibitors that are involved in releasing BCR-ABL+ from the bone marrow niche might increase responsiveness to TKIs. In CML, miRNA expression profile has a potential role as an effective diagnostic biomarker, monitoring disease progression and drug response. Furthermore, a biological understanding of the role of the miRNA in TKIs response and resistance still needs to be researched.

## Data availability

No data are associated with this article.
