# Supplementary material for: Contribution of BCR-ABL molecular variants and leukemic stem cells in response and resistance to tyrosine kinase inhibitors: a review
Source: F1000Res. 2022 Feb 9;10:1288. Originally published 2021 Dec 15. [Version 2] doi: 10.12688/f1000research.74570.2 (PMC8886173; doi:10.12688/f1000research.74570.2)
Supplement: Peer review history

# Peer review: Reviewer response for version 2

**DOI:** 10.5256/f1000research.120985.r125621

**Published:** 2022-02-28

**Authors:** Pinar Arslan

**Affiliations:** 1 Biology Department, Faculty of Science, Çankırı Karatekin University, Çankırı, Turkey

The review is about chronic myeloid leukemia (CML) connections with stem cells. First, the BCR-ABL gene locus is mentioned in the chromosome and then proteins are described. During the treatment of CML, TKI has been changed. TKI mainly target the BCR-ABL genes. The CML stem cells are some subpopulations of CML cells and are found in the patients’ tissues in the diagnosis and treatment procedure. Therefore, CML is introduced very well. Besides, most of the figures are clear. The author has made the corrections suggested by the reviewer. However, the manuscript may be enhanced by the below points:

1. In the introduction, the author is recommended to add information regarding other types of Leukemias with BCR-ABL translocation.
2. Figure 2 is not clear to me. I would replace it with a table clarifying the various mutations and their responses to TKIs.
3. I would suggest the English revision of the article.

Is the review written in accessible language?

Yes

Are all factual statements correct and adequately supported by citations?

Yes

Are the conclusions drawn appropriate in the context of the current research literature?

Yes

Is the topic of the review discussed comprehensively in the context of the current literature?

Yes

Reviewer Expertise:

Cell biology

I confirm that I have read this submission and believe that I have an appropriate level of expertise to confirm that it is of an acceptable scientific standard.

# Peer review: Reviewer response for version 2

**DOI:** 10.5256/f1000research.120985.r123029

**Published:** 2022-02-23

**Authors:** Ala-Eddin Al Moustafa

**Affiliations:** 1 College of Medicine, QU Health, Qatar University, Doha, Qatar

The authors addressed all my comments.

Is the review written in accessible language?

Yes

Are all factual statements correct and adequately supported by citations?

Yes

Are the conclusions drawn appropriate in the context of the current research literature?

Yes

Is the topic of the review discussed comprehensively in the context of the current literature?

Yes

Reviewer Expertise:

Yes

I confirm that I have read this submission and believe that I have an appropriate level of expertise to confirm that it is of an acceptable scientific standard.

# Peer review: Reviewer response for version 1

**DOI:** 10.5256/f1000research.78343.r118639

**Published:** 2022-01-24

**Authors:** Ala-Eddin Al Moustafa

**Affiliations:** 1 College of Medicine, QU Health, Qatar University, Doha, Qatar

In this manuscript, the author reviewed the role of the BCR-ABL gene and its inhibitor (TKI therapy) in the response and resistance of Chronic myeloid leukemia (CML). This is an interesting review paper that can be accepted for publication after some revisions as follows:

1. The author should start the introduction with a paragraph about leukemia in general and its different categories, including CML.
2. The author should remove Figure 1 from the paper which is not necessary.
3. The author should replace Figure 2 with a table.
4. In the conclusion section, “miRNA expression profile has a potential role as an effective diagnostic biomarker” the word “expression” should be removed and replaced by “miRNA profile, particularly circulating …”
5. The paper needs some revision for English.

Is the review written in accessible language?

Yes

Are all factual statements correct and adequately supported by citations?

Yes

Are the conclusions drawn appropriate in the context of the current research literature?

Yes

Is the topic of the review discussed comprehensively in the context of the current literature?

Yes

Reviewer Expertise:

Yes

I confirm that I have read this submission and believe that I have an appropriate level of expertise to confirm that it is of an acceptable scientific standard, however I have significant reservations, as outlined above.

# Response

**Published:** 2022-02-07

**Authors:** Mohammad Al Hamad

**Affiliations:** Imam Abdulrahman bin Faisal University, Saudi Arabia

1. A paragraph about leukemia in general and its different categories is added.
2. Figure 1 is removed from the paper.
3. Figure 2 is replaced with a table.
4. “expression” is removed and replaced by “miRNA” profile.
5. Revision for English is done.
